# Association of L-Ficolin Levels and *FCN2* Genotypes with Chronic Chagas Disease

**DOI:** 10.1371/journal.pone.0060237

**Published:** 2013-04-04

**Authors:** Paola R. Luz, Angelica B. W. Boldt, Caroline Grisbach, Jürgen F. J. Kun, Thirumalaisamy P. Velavan, Iara J. T. Messias-Reason

**Affiliations:** 1 Laboratório de Imunopatologia Molecular – Departamento de Patologia Médica, Hospital de Clínicas, Universidade Federal do Paraná, Curitiba, Brazil; 2 Institute of Tropical Medicine, University of Tuebingen, Tuebingen, Germany; University Medical Center Utrecht, The Netherlands

## Abstract

**Background:**

L-ficolin (encoded by *FCN2*) binds to acetylated sugar moieties of many pathogens, including *Trypanosoma cruzi*, promoting their phagocytosis and lysis by the complement system.

**Methods:**

We investigated L-ficolin levels in 160 *T. cruzi* infected patients with chronic Chagas disease and 71 healthy individuals, and *FCN2* polymorphisms (−*986 G>A*, *−602 G>A*, and *−4 A>G* in the promoter and *A258S* in exon 8) in 243 patients, being 88 indeterminate (asymptomatic), 96 with cardiac, 23 with digestive and 33 with cardiodigestive manifestations (two were unspecified) and 305 controls (135 for *A258S*).

**Results:**

Patients presented lower L-ficolin plasma levels than controls (p<0.0001). Among the different groups of cardiac commitment, individuals with moderate forms had higher L-ficolin levels than the severe forms (P = 0.039). Lower L-ficolin levels were found associated with the *258S* variant in the patients (P = 0.034). We found less *−4A/G* heterozygotes in the cardiac patients, than in the controls (OR = 0.56 [95% CI = 0.33–0.94], P = 0.034). Heterozygote *−4A/G* genotypes with the *258S* variant and *258SS* homozygotes were nevertheless more frequent among cardiodigestive patients than in controls (OR = 14.1 [95% CI = 3.5–56.8], P = 0.0001) and in indeterminate patients (OR = 3.2 [95% CI = 1.1–9.4], P = 0.037). We also found an association of the allelic frequency of the *258S* variant with cardiodigestive Chagas disease compared to controls (OR = 2.24 [95% CI = 1.1–4.5], P = 0.037). Thus, decreased patient levels of L-ficolin reflect not only protein consumption due to the disease process, but also the higher frequency of the *258S* variant in patients with cardiodigestive symptoms.

**Conclusion:**

The very first study on Brazilian cohort associates both L-ficolin plasma levels and *FCN2* variants to Chagas disease and subsequent disease progression. The prognostic value of L-ficolin levels and the FCN2*A258S polymorphism should be further evaluated in other settings.

## Introduction

Chagas disease (CD) occurs in 18 different countries, mostly throughout South and Central America, and affects approximately 15 million people worldwide [1]. CD pathogenesis, caused by the flagellated protozoan *Trypanosoma cruzi*, is still poorly understood and there is no available marker that indicates the progression to the different clinical forms or prognosis of chronic disease. Despite the fact that approximately 50% of the individuals infected by *T. cruzi* stay in the indeterminate or asymptomatic form throughout their lives, which in general present good prognosis, each year about 2–5% of them progress to symptomatic forms of the disease, presenting irreversible cardiac and/or digestive and/or disorders [2]. A plausible presumption is that individuals who remain asymptomatic are able to reduce parasite numbers in the early phase of infection, and down modulate the immune response, limiting the development of pathology. On the other hand, individuals who will develop cardiac disease, although capable of parasite control, may not be capable of mounting efficient immunoregulatory mechanisms, thus leading to establishment of persistent inflammation [3].

The complement system has been shown to play an important role in the control of experimental *T. cruzi* infection, as well in clinical evolution of CD [4]–[8]. Ficolins are pattern-recognition proteins which bind to specific pathogen-associated molecular patterns (PAMP) on microorganism surfaces, promoting activation of the complement cascade through the lectin pathway thereby triggering the innate immune response [9]. Other putative functions of ficolins include binding to late apoptotic cells, apoptotic bodies and necrotic cells, enhancing their uptake by macrophages [10]. The ficolins are synthesized as a single polypeptide containing N-collagen-tails and C-terminal fibrinogen-like binding domains, which are oligomerized into higher oligomeric forms [9]. In humans, three ficolin genes have been identified: *FCN1*, *FCN2* and *FCN3*, which encode M-ficolin (ficolin-1), L-ficolin (ficolin-2) and H-ficolin (Hakata antigen or ficolin-3), respectively. Single nucleotide polymorphisms (SNPs) in the promoter region of the *FCN2* gene have been associated with the variability in the individual serum concentrations of the protein. Studies have shown that the presence of the nucleotide adenine (*A*) at positions −986 A>G and −602 A>G as well as the nucleotide guanine (*G*) at the position −4G>A are related to higher L-ficolin serum levels [11]. In addition, two polymorphisms located in exon 8, encoding the fibrinogen-like domain (containing *T236M* and *A258S*) are associated with decreased and increased ability of binding to acetylated residues, respectively [11]–[13]. L-ficolin levels and *FCN2* polymorphisms have been reported to be associated with different diseases [14]–[20].

Both L-ficolin and H-ficolin are able to interact with the mannan-binding lectin (MBL)-associated serine proteases, promoting activation of the complement cascade [21]. L-ficolin recognizes N-acetylated molecules, including N-acetylglucosamine (GlcNAc) or N-acetylgalactosamine (GalNAc) [22], [23]. The surface of *T. cruzi* contains a large diversity of N-linked and O-linked carbohydrate-rich molecules and it was recently demonstrated that *T. cruzi* activates the lectin pathway [7]. The authors have shown that a fast binding of L-ficolin occurs on the surface of *T. cruzi* and that the depletion of these molecules in the serum leads to failure of parasite clearance by the complement, indicating that the lectin pathway obviously plays an important role in host defense against this pathogen. Although it has been demonstrated that L-ficolin is involved in the host defense against *T. cruzi* infection, there are no studies on ficolins and chronic CD so far. In this study we aim to evaluate whether L-ficolin levels and *FCN2* polymorphisms could be possible prognostic markers for susceptibility to the different clinical forms of CD.

## Materials and Methods

### Ethics Statement

Formal written consent approved by the ethics committee was obtained from each individual. The project was approved by the ethics committee of Hospital De Clinicas, Universidade Federal do Parana (CEP/HC-UFPR n.1457.122/2007-06).

### Subjects and Samples

A total of 243 chronic CD patients attended at the Chagas Disease Ambulatory of the Clinical Hospital of the Federal University of Paraná (HC- UFPR) were investigated (Mean age 57.3 [34–90] years; 58% female, 42% male; 76.5% Euro-Brazilian, 18.9% Afro-Brazilian, 4.1% Amerindian, 0.5% Asian). CD diagnosis was performed by serological and clinical examinations. The clinical history of the patients was obtained from medical records and interviews, using a standard questionnaire. Patients younger than 18 years-old, with a history of blood transfusion, recent infections or suspected non-chagasic cardiomyopathy (such as hypertensive cardiopathy) were excluded. The cardiac patients were graded according to the cardiac insufficiency classification of the American Heart Association (AHA), adapted for Chagas disease [24]. Detailed demographic and clinical characteristics of the specific CD forms are given in Table 1. A group of 305 unrelated South Brazilians with negative Chagas (anti-*Trypanosoma cruzi*) serology and without non-chagasic cardiomyopathy were used as controls (Mean age 41 [18–75] years; 49.2% female, 50.8% male; 85.2% Euro-Brazilian, 12.8% Afro-Brazilian, 1.3% Amerindian, 0.7% Asian). Ethnic background of patients and controls was determined as previously described [25].

**Table 1:** Demographic and clinical parameters of chronic Chagas patients.

Parameters	Chagas form	Indeterminate	Cardiac	Digestive	Cardiodigestive
	N	88	96	23	33
Age	Average:	54.6	58.9	59.3	58.2
(years)	[Min-Max]	[34–76]	[34–90]	[36–84]	[37–73]
Gender	Female:	38.6	50.0	78.3	42.4
(% )	Male:	61.4	50.0	21.7	57.6
Ethnic group	European:	87.5	67.7	65.2	78.8
(%)	African:	9.1	26.0	30.4	18.2
	Asian:	0	1.0	4.3	0
	Amerindian:	3.4	5.2	0	3
Class of cardiac Commitment & (%)	A:	3.4	24.0	N.d.	33.3
	B1:	2.3	25.0	N.d.	21.2
	B2:	0	3.1	N.d.	0
	C:	1.1	36.4	N.d.	33.3
	D:	0	20.8	N.d.	6.1
Left ventricular ejection fraction (%)	(N) Average: [Min-Max]	(47) 132.8 [35–78]	(79) 58 [24–84]	N.d.	(31) 55.1 [23–81]
C-reactive protein* mg/dl	(N) Average: [Min-Max]	(28) 0.74 [0.08–3.77]	(47) 0.66 [0.08–4.25]	(4) 0.19 [0.09–0.31]	(11) 0.33 [0.08–0.76]
Mannose-binding lectin* ng/dl	(N) Average: [Min-Max]	(53) 3514 [42–6379]	(58) 2270 [50–7214]	(15) 1909 [50–5465]	(20) 2222 [50–5600]

* MBL levels were determined by ELISA and C-reactive protein (hs-CRP) by nephelometry as previously described [6] n number of investigated samples; N.d. not determined.

& some patients were unspecified.

After signing the informed formal written consent, three ml of venous blood from each individual was collected in tubes containing ethylenediamine tetra-acetic acid (EDTA). Samples were maintained on ice after collection and during transportation to the laboratory. After centrifugation at −4°C plasma separation from whole blood was performed as quickly as possible, samples were aliquoted on ice and immediately stored at −80°C until used. Genomic DNA was extracted from peripheral whole blood using commercial kits (GFX™ Genomic Blood DNA Purification Kit, GE Healthcare, São Paulo, Brazil), according to the manufacturer’s instructions.

### L-fciolin Measurement

The quantification of plasma L-ficolin levels was performed in 71 controls (of which 62 were genotyped) and 160 patients (of which 158 were genotyped, being 54 indeterminate, 54 with some kind of cardiomyopathy, 19 with digestive symptoms, 29 with the cardiodigestive form and two unspecified). L-ficolin levels were determined using a commercially available enzyme linked immunosorbent assay (ELISA) kit (HK336, Hycult® Biotech, Uden, Netherlands), according to the manufacturer’s instructions. The detecting range of this kit is 16–1000 ng/ml of the protein.

### Genotyping of *FCN2* Variants

Three promoter SNPs including *−986 G>A* (rs3124952), *−602 G>A* (rs3124953), *−4 A>G* (rs17514136) and *A258S* in exon 8 (rs7851696) were genotyped in the patients using Fluorescence Resonance Energy Transfer (FRET) based real-time PCR assay as previously described [26], and in 135 controls through sequencing [14]. Additionally, 170 patients were genotyped for the SNPs −986 G>A (rs3124952), −602 G>A (rs3124953) and −4 A>G (rs17514136) using PCR amplification with sequence-specific primers (SSP) (Table 2). In order to reconstruct the haplotypes, we performed three reactions called FCN2 Prom12, FCN2 Prom23 and FCN2Prom13, using the primers listed in table 2. In order to validate the reactions, we added two generic primers, either HGHf and HGHr or FCN2Ex8f and FCN2Ex8r. PCR amplifications were carried out in a Therm-2000 (Axygen, United States) or MJ96+ (Biocycler, China) research thermocyclers with 20 ng DNA, 0.2 mM dNTP (Invitrogen Life Technologies, United States), 0.2 µM of each primer (Invitrogen Life Technologies, United States), 1X Coral Load PCR buffer (Qiagen, The Netherlands), 2 mM of MgCl2 (Invitrogen Life Technologies, United States), 0.35–0.7 mM of Q solution (Invitrogen Life Technologies, United States), and 0.15 U Taq DNA polymerase (Invitrogen Life Technologies or Promega, United States) in a final volume of 15 µl. After a 5 min initial DNA denaturation step at 94°C, the PCR protocol was followed by 30 cycles of 30 sec at 94°C, 45 sec at the specific annealing temperature and 40 sec at 72°C, ending with 5 min at 72°C in the final DNA extension step. A touch-down strategy decreasing the annealing temperatures every 10 cycles was performed, using 68°C-64°C-62°C for FCN2 Prom12, 65°C-62°C-59°C for FCN2 Prom23 and 69°C-67°C-65°C for FCN2Prom13. According to a previous study, this strategy assures higher specificity to the amplification and provides a larger amount of the desired PCR product [27]. The promoter haplotypes amplified by a pair of SSPs were identified by the presence or absence of specific bands when run on a 1.2% agarose gel electrophoresis. Positive controls reassured the quality of the reactions.

**Table 2:** Primers used in the sequence-specific PCR (PCR-SSP) for the −986, −602 and −4 promoter *FCN2* polymorphisms.

Forward Primer		Reverse primer		size
PCR-SSP Prom1,2				
*FCN2* Prom −986 Af	5′ ACCTCGGCATCCCGATGGCA 3′	FCN2 Prom −602 Ar	5′ TATGTAGAGCACAGGGGCACAT 3′	374 bp
*FCN2* Prom −986 Gf	5′ ACCTCGGCATCCCGATGGCG 3′	FCN2 Prom −602 Gr	5′ TATGTAGAGCACAGGGGCACAC 3′	374 bp
PCR-SSP Prom2,3				
*FCN2* Prom −602 Af	5′ TCTCTCCTTTCCCTCCTGTTCA 3′	FCN2 Prom −4 Ar	5′ GCTCTGTCCAGCTCCATCTCT 3′	648 bp
*FCN2* Prom −602 Gf	5′ TCTCTCCTTTCCCTCCTGTTCG 3′	FCN2 Prom −4 Gr	5′ GCTCTGTCCAGCTCCATCTCC 3′	648 bp
PCR-SSP Prom1,3				
*FCN2* Prom −986 Af	5′ ACCTCGGCATCCCGATGGCA 3′	FCN2 Prom −4 Ar	5′ GCTCTGTCCAGCTCCATCTCT 3′	1022 bp
*FCN2* Prom −986 Gf	5′ ACCTCGGCATCCCGATGGCG 3′	FCN2 Prom −4 Gr	5′ GCTCTGTCCAGCTCCATCTCC 3′	1022 bp
Endogenous control				
HGH f	5′ TGCCTTCCCAACCATTCCCTTA 3′	HGH r	5′ CCACTCACGGATTTCTGTTGTGTTTC 3′	431 bp
FCN2 Ex8f	5′ GCCAGGCCTCAGGTATAAAG 3′	FCN2 Ex8r	5′ AAAGGGTTGATTGCGGAAAC 3′	500 bp

Primer design was based on the NT_019501 reference sequence. FCN2– Ficolin 2; HGH – Human Growth Hormone; f – forward; r – reverse; In bold: variant nucleotides; bp – base pairs; Prom – promoter; Ex8– exon 8.

### Statistical Analysis

Statistics was done using the statistical package for social sciences (SPSS) version 10.0, STATA ver 9.1 and with the Graphpad Prism 5.04 software package. Data were normalized and have been analyzed by STATA. Genotype and allele frequencies were obtained by direct counting. The hypothesis of Hardy–Weinberg equilibrium and of homogeneity between genotype distributions (exact test of population differentiation of Raymond and Rousset) were executed with the ARLEQUIN software package version 3 [28]. Possible associations between *FCN2* genotypes/haplotypes/variants and different clinical forms were analyzed with two tailed Fisher’s exact test. Additionally multivariate analysis was executed to validate whether variables such as age, gender and ethnicity may possibly influence the clinical outcome by MANOVA. L-ficolin concentrations in the different groups were presented by the median and range and compared between the genotypes using either nonparametric Mann-Whitney or Kruskal-Wallis tests. Also correlation between L-ficolin levels to age was tested using spearman’s rank correlation. Unless otherwise stated, two-tailed P-values less than 5% were considered significant. *P-*values for correlations are provided after correction for false detection rate (FDR).

## Results

### L-ficolin Levels

**Figure 1:**
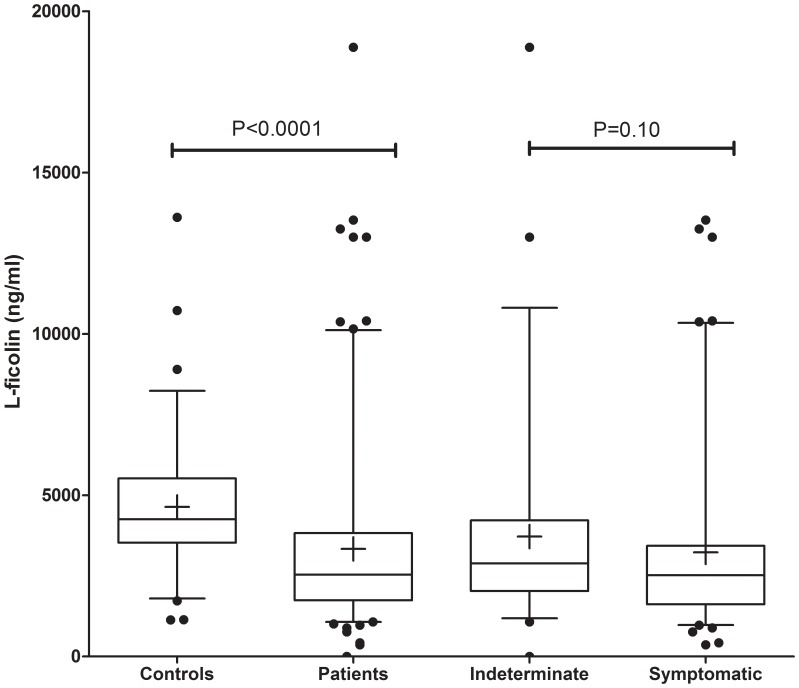
L-ficolin levels in Chagas patients and controls. The statistical distribution is shown with median (line in the box), box indicating the 25–75 percentiles, whiskers the 5–95 percentile and arithmetic mean (cross inside the box).

**Figure 2:**
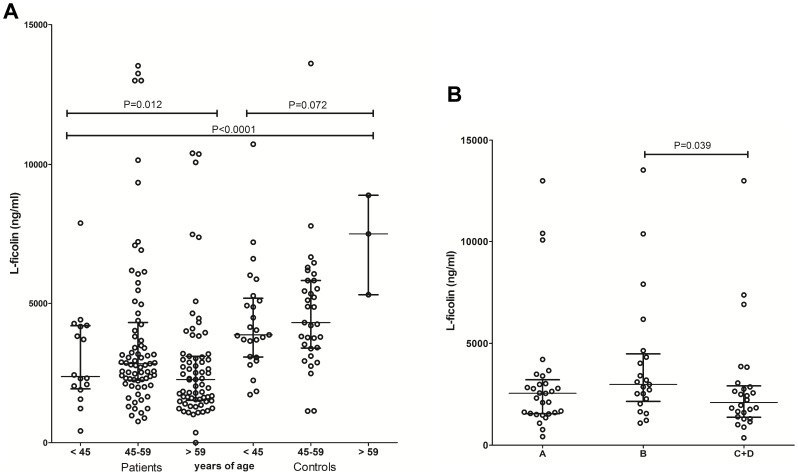
Distribution of L-ficolin levels based on age and clinical classification. (A) L-ficolin levels in Chagas patients and controls according to age (B) L-ficolin levels in Chagas patients and controls based on class of cardiac commitment. The statistical distribution is shown with median and interquartile range. One outlier (18880.7 ng/ml) was excluded for better visualization in the 45–59 group of patients.

Higher L-ficolin plasma concentrations were observed in the controls when compared to Chagas patients (median: 4252 ng/ml vs. 2538 ng/ml, P<0.0001). Similar trend was observed between indeterminate and symptomatic patients (median: 2888 ng/ml vs. 2519 ng/ml, one-tailed P = 0.051, two-tailed P = 0.10) (Figure 1). No significant correlation between L-ficolin levels and age has been observed using Spearman’s rank correlation test (Spearman’s rho = −0.1402, p = 0.08). Nevertheless L-ficolin levels were higher in the group with 45 to 59 years of age, compared with those aged above 60 years (median: 2834 ng/ml vs. 2265 ng/ml, P = 0.005). Nevertheless they were still lower than the levels found in the 45–59 years group of controls, which had a median of 4311 ng/ml (P = 0.0007) (Figure 2). There was also a trend to increased L-ficolin concentration in the healthy adults (P = 0.072). Among the different groups of cardiac commitment, individuals with the less severe B form had higher L-ficolin levels than the C and D forms (median: 2981 ng/ml vs. 2094 ng/ml, P = 0.039) (Figure 2). Among the clinical parameters (listed in Table 1), only mannose-binding lectin correlated weakly with L-ficolin levels in the indeterminate patients (R2 = 0.38, P = 0.004), but not in the other disease stages (not shown). Tests for multivariate analysis revealed no significant effects of age, gender or ethnicity to L-ficolin levels.

### *FCN2* Polymorphisms

Five haplotypes comprehending the −*986 G>A,* −*602 G>A,* −*4 A>G* variants in the promoter and the amino acid substitution *A258S* in exon 8 were identified: *AAAA*, *AGAA*, *AGGA, GGAA* and *GGAS* (Table 3). Haplotype distributions did not differ between the different group and genotype distributions were in Hardy Weinberg equilibrium. Nevertheless we observed less −*4A/G* heterozygotes (comprehending the promoter genotypes *AAA/AGG, AGA/AGG* and *AGG/GGA*) in the cardiac patients than in the controls (23/96 or 24% vs. 110/305 or 36.1%, OR = 0.56 [95% CI = 0.33–0.94], P = 0.034), and there was also a trend in the same direction between cardiac and indeterminate patients (P = 0.077) (Table 3). Heterozygote −*4A/G* genotypes with the *258S* variant in exon 8 (*AGGA/GGAS*) and *258S* homozygotes (*GGAS/GGAS*) were more frequent among cardiodigestive patients, than in controls (8/33 or 24.2% vs. 3/135 or 2.22%, respectively, OR = 14.1 [95% CI = 3.5–56.8], P = 0.0001) and in indeterminate patients (8/33 or 24.2% vs. 8/88 or 9.1%, OR = 3.2 [95% CI = 1.1–9.4], P = 0.037). We also found an association of the allelic frequency of the *258S* variant with cardiodigestive Chagas disease (14/66 or 21.2% vs. 29/270 or 10.7% in the controls, OR = 2.24 [95%CI = 1.1–4.5], P = 0.037) (Tables 3 and 4).

**Table 3:** *FCN2* allele and haplotype frequencies (± standard deviation) in Chagas patients and controls.

FCN2N	Controls610	Patients486	Indeterminate176	Symptomatic304	Cardiac192	Digestive46	Associated66
−986 G>A	50.0±2.5	43.4±2.3	46.0±3.8	41.4±2.8	40.6±3.6	43.5±7.4	42.4±6.1
−602 G>A	19.3±2.0	17.9±1.7	19.3±3.0	16.8±2.1	16.7±2.7	15.2±5.4	18.2±4.8
−4 A>G	23.4±2.2	20.6±1.8	20.5±3.0	20.7±2.3	19.3±2.9	23.9±6.4	22.7±5.2
A258S	10.7±1.9*	15.2±1.6	13.6±2.6	16.1±2.1	14.6±2.6	15.2±5.4	21.2±5.1
AAAA	19.3±2.0	17.9±1.7	19.3±3.0	16.8±2.1	16.7±2.7	15.2±5.3	18.2±4.8
AGGA	23.4±2.2	20.6±1.8	20.5±3.0	20.7±2.3	19.3±2.9	23.9±6.4	22.7±5.2
AGAA	7.3±1.3	4.9±1.0	6.3±1.8	3.9±1.1	4.7±1.5	4.3±3.0	1.5±1.5
GGA	52.0±2.0	56.6±2.3	54.0±3.8	58.6±2.8	59.4±3.6	56.5±7.4	57.6±6.1
GGAA	41.1±3.0	41.4±2.2	40.3±3.7	42.4±2.8	44.8±3.6	41.3±7.3	36.4±6.0
GGAS	10.7±1.9*	15.2±1.6	13.6±2.6	16.1±2.1	14.6±2.6	15.2±5.4	21.2±5.1

In bold: significant difference (see text).

* *A258S* was investigated by sequencing in a subset of 135 samples. N number of chromosomes.

**Table 4:** *FCN2* genotype frequencies (%) in Chagas patients and controls.

FCN2N	Controls305	Patients243	Indeterminate88	Symptomatic152	Cardiac96	Digestive23	Associated33
AAAA/AAAA	10 (3.3)	6 (2.5)	3 (3.4)	3 (2.0)	2 (2.1)	0	1 (3.0)
AAAA/AGAA	6 (2.0)	7 (2.9)	3 (3.4)	4 (2.6)	3 (3.1)	0	1 (3.0)
**AAAA/AGGA**	**22 (7.2)**	14 (5.8)	7 (8.0)	6 (4.0)	**3 (3.1)**	2 (8.7)	1 (3.0)
AAAA/GGAA	20 (14.8)*	36 (14.8)	11 (12.5)	25 (16.4)	15 (15.6)	4 (17.4)	6 (18.2)
AAAA/GGAS	7 (5.2)*	18 (7.4)	7 (8.0)	10 (6.6)	7 (7.3)	1 (4.3)	2 (6.1)
AAA/GGA	57 (18.7)	54 (22.2)	18 (20.5)	35 (23.0)	22 (22.9)	5 (21.7)	8 (24.2)
AGAA/AGAA	0	1 (0.4)	1 (1.1)	0	0	0	0
**AGAA/AGGA**	**9 (3.0)**	4 (1.6)	3 (3.4)	1 (0.7)	**1 (1.0)**	0	0
AGAA/GGAA	7 (5.2)*	9 (3.7)	1 (1.1)	7 (4.6)	5 (5.2)	2 (8.7)	0
AGAA/GGAS	2 (1.5)*	2 (0.8)	2 (2.3)	0	0	0	0
AGA/GGA	27 (8.9)	11 (4.5)	3 (3.4)	7 (4.6)	5 (5.2)	2 (8.7)	0
AGGA/AGGA	18 (5.9)	12 (4.9)	2 (2.3)	10 (6.6)	7 (7.3)	2 (8.7)	1 (3.0)
AGGA/GGAA	31 (23.0)*	36 (14.8)	15 (17.0)	21 (13.8)	12 (12.5)	3 (13.0)	6 (18.2)
**AGGA/GGAS**	**3 (2.2)** *	22 (9.1)	**7 (8.0)**	15 (9.9)	7 (7.3)	2 (8.7)	**6 (18.2)**
**AGG/GGA**	**79 (25.9)**	58 (23.9)	22 (25.0)	36 (23.7)	**19 (19.8)**	5 (21.7)	12 (36.4)
GGAA/GGAA	18 (13.3)*	47 (19.3)	19 (21.6)	28 (18.4)	20 (20.8)	3 (13.0)	5 (15.2)
GGAA/GGAS	17 (12.6)*	26 (10.7)	6 (6.8)	20 (13.2)	14 (14.6)	4 (17.4)	2 (6.1)
GGAS/GGAS	**0** *	3 (1.2)	**1 (1.1)**	2 (1.3)	0	0	**2 (6.1)**
GGA/GGA	77 (25.2)	76 (31.3)	26 (29.5)	50 (32.9)	34 (35.4)	7 (30.4)	9 (27.3)

In bold: genotypes with −*4 A/G* heterozygosity, whose summed frequencies differ between cardiac patients and controls. Gray-shadowed: genotypes *AGGA/GGAS* and *GGAS/GGAS* (homozygote for *A258S*), whose summed frequencies differ between cardiodigestive patients and indeterminate patients or controls (see text).

* *A258S* was investigated by sequencing in a subset of 135 controls. n number of individuals.

### Association of L-ficolin Levels with *FCN2* Genotypes

**Figure 3:**
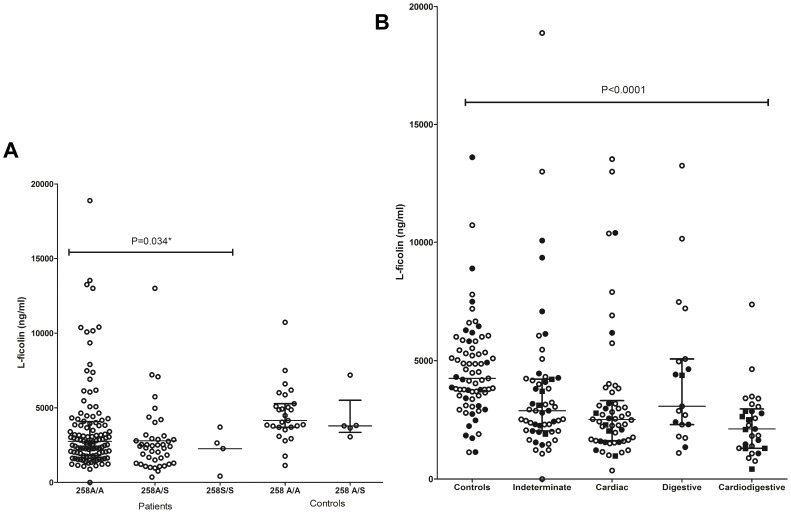
Distribution of L-ficolin levels based on genotypic variant and disease stages. (A) L-ficolin levels in Chagas patients and controls by the presence of *258S* variant and (B) Distribution of L-ficolin levels in Chagas patients and controls by disease stages. The statistical distribution is shown with median and interquartile range. Closed dots: samples with −*4 A/G* genotype. Closed boxes: samples with −*4 A/G* and *258A/S* (*AGGA/GGAS* genotype) or *258S/S* homozygotes (*GGAS/GGAS* genotype).

Lower L-ficolin levels were found associated with the *258S* variant in the patients (median: 2419 ng/ml vs. 2748 ng/ml in genotypes without *258S*, P = 0.034). There was no difference between the levels according to the other genotypes, even in the controls (not shown). Whereas heterozygote genotypes with −*4 A/G* were, in general, widely distributed according to L-ficolin levels in the different groups, those with *258 S* (genotype *AGGA/GGAS*) as well as *258 S/S* (*GGAS/GGAS*) homozygotes, clearly associated with concentrations under 3000 ng/ml (Wilcoxon signed rank test, P = 0.04) (Figure 3).

## Discussion

In this study, L-ficolin levels were measured in plasma of the Chagas patients and controls. L-ficolin levels in the controls were within the range reported for healthy European adults in the early studies [29], [30]. A decreased L-ficolin plasma level were observed in the Chagas patients when compared to controls, and a similar trend was observed between symptomatic patients and indeterminate, however, this difference was not significant. Importantly, serum L-ficolin concentrations were also lower in Bronchiectasis patients than controls from the UK [29]. In contrast to our previous work with the same patients, where MBL levels increased gradually according to age, L-ficolin levels were higher in the group with 45 to 59 years of age, compared with those aged above 60 years. There was also a trend to increased L-ficolin concentration in the healthy adults (P = 0.072), but others did not find any relationship between L-ficolin concentration and age in healthy Scottish adults [29]. There was no significant difference between L-ficolin levels according to the gender of patients and controls, as expected [29]. In addition, the difference of L-ficolin levels was observed among the different groups of cardiac commitment. Of which individuals with the less severe B form had higher L-ficolin levels than the C and D forms. Interestingly, median L-ficolin levels are higher in acute than in chronic hepatitis B conditions [15]. They are also higher in acute malaria, compared with the levels after treatment [16], but lower in *Schistosoma haematobium*-infected individuals from Nigeria, compared to controls [17]. Our results contrast with those found by our group in the same patients regarding MBL levels, which were much higher (above 1000 ng/ml) in patients with chagasic cardiomyopathy and did not differ between the B C and D stages [6]. Furthermore, a weak correlation between mannose-binding lectin and L-ficolin levels was observed in the indeterminate patients, but not in the other disease stages.

Allele and haplotype frequencies in the controls did not differ from those reported for European-derived populations [11], [26]. No significant difference of haplotype distribution was observed between different groups, nevertheless, −*4 A/G* heterozygotes was observed to associate with the disease when compared cardiac patients to the controls as well as cardiac to the indeterminate patients. In addition, the *AGG* promoter haplotype, whose presence is obligatory in −*4 A/G* heterozygotes, was also associated with protection against HBV infection in Vietnamese [15], but with susceptibility to schistosomiasis in Nigeria [17]. Our group found the *AGA* haplotype to be associated with protection against clinical leprosy and rheumatic fever [14], [18], whereas others found it associated with susceptibility to cutaneous leishmaniasis [28]. Ficolin-2 seems to be, as MBL, a double-edged sword in immunity, explaining the contrasting results in association studies with different infectious diseases. We also found an association of the allelic frequency of the *258S* variant with cardiodigestive Chagas disease (Tables 3 and 4). The *258S* variant was also found associated by others with earlier onset of *Pseudomonas aeruginosa* colonization in cystic fibrosis patients [31]. In contrast to our findings, homozygosity for this variant was associated with protection against cutaneous leishmaniasis in Syria [32] and the presence of this allele, with protection against cytomegalovirus, but not bacterial infections, after orthotopic liver transplantation [33], [34]. It was not associated with respiratory tract infections in Dutch children [35], nor with invasive pneumococcal disease [36], rheumatoid arthritis [37] and Behçet’s disease in Japan [38]. Interestingly, FCN2*258S, also known as *FCN2-C*, has increased N-acetyl-D-glucosamine (GlcNAc) binding capacity [11] and *O*-linked GlcNAc moieties constitute a common epitope between cruzipain, a major *T. cruzi* antigen, and either myosin or other cardiac O-GlcNAc-containing proteins [39]. Higher GlcNAc binding capacity of this variant may thus correlate with complement activation and destructive deposition of membrane attack complexes (MAC) on muscle tissues of patients with cardiodigestive manifestations. At least for chronic chagasic cardiomyopathy, increased MAC deposition has been observed, in contrast to patients with idiopathic dilated cardiomyopathy or controls [40]. Additionally, our study also showed that there are associations between 258S variant and L-ficolin level in the patients. The association between low L-ficolin levels and the *258S* variant has also formerly been observed in other settings: healthy Danish individuals [13], Polish children with recurrent infections [12], Gabonese malaria patients (but not controls) [16], Vietnamese HBV-infected patients and controls [15].

In conclusion, this is the first report on L-ficolin levels and *FCN2* polymorphisms in patients with chronic CD. Activation of the lectin and alternative complement system is a crucial defense mechanism for the control of *T. cruzi* parasitemia [7] and L-ficolin has been shown to bind metacyclic trypomastigotes [7]. Decreased levels of L-ficolin in chagasic patients reflect, in part, the higher frequency of the *258S* variant in those with cardiodigestive symptoms. However, since most of the *FCN2* genotypes did not associate with L-ficolin levels, reduction may also be due to the disease process itself, since L-ficolin can bind to *T.cruzi* antigens [7], to pentraxin 3 [41] and to the C-reactive protein [42], which might affect circulating levels. In addition, inflammatory activity and the resulting tissue fibrosis could be further exacerbated by the higher capacity of GlcNAc binding to the *258S* variant. L-ficolin levels and consumption could thus be used as markers of disease activity, but further experiments should be performed to prove this hypothesis.
